# ‘A really good balance’: Thematic analysis of stakeholders’ views on classroom- and games-based positive choices interventions for primary school children

**DOI:** 10.1371/journal.pone.0219503

**Published:** 2019-07-09

**Authors:** Nicola McCullogh, Spencer E. Boyle, Melissa Fothergill, Margaret Anne Defeyter

**Affiliations:** 1 Department of Sport, Exercise and Rehabilitation, Northumbria University, United Kingdom; 2 School of Psychology, Newcastle University, United Kingdom; 3 Department of Psychology, Northumbria University, United Kingdom; Teesside University/Qatar Metabolic Institute, UNITED KINGDOM

## Abstract

This study explores the views of children, parents, school staff and intervention staff regarding interventions designed to promote healthy lifestyles and positive choices in primary schools in the North East of England, United Kingdom. The interventions consisted of six weekly sessions in which classroom learning was followed by physically active games. Focus groups and semi-structured interviews were conducted with a total of 45 participants and thematic analysis was performed on the resultant 26 transcripts to identify themes relating to the role of physical activity, facilitators and barriers to children’s engagement in the sessions and the perceived outcomes of intervention participation. Results indicated that participants across the four groups felt the inclusion of classroom learning and physical activity made the interventions suitable for a range of children, with the games reinforcing classroom messages and acting as a reward for their work. Central to children’s active engagement was their enjoyment, and they were felt to benefit in terms of psychosocial wellbeing and–especially when the topic of the intervention was fitness and nutrition–physical wellbeing. Overall, combined classroom- and games-based interventions were valued methods for communicating healthy lifestyle and positive choices messages to a primary school audience, though research into intervention outcomes is currently limited.

## Introduction

While not a statutory subject, the national curriculum in England states that schools “should make provision for personal, social, health and economic education (PSHE)” [1]. The Department for Education advises that schools can tailor their PSHE programmes to the needs of their pupils but should provide learners with an “understanding of risk and with the knowledge and skills necessary to make safe and informed decisions” [2]. Suggested topics for upper-primary school children (ages 7–11 years) include maintaining a healthy lifestyle via diet and exercise, understanding emotions, developing an awareness of drugs and recognising the effects of bullying and discrimination [3]. Not only have improvements been found in the social and emotional skills of young people following their participation in programmes that aim to develop their self-awareness, social awareness and responsible decision making, but gains in academic achievement have also been demonstrated via increased school grades or scores on standardised achievement tests for reading and mathematics [4]. The outcomes of physical activity (PA) participation complement those of PSHE programmes, with improvements having been noted for self-esteem, self-discipline, teamwork/social inclusion, responsibility and assertiveness in addition to physical health [5].

Despite the above outcomes, it is widely acknowledged that core curriculum subjects such as English and mathematics are given priority in the school day [6], with time allocated for lessons such as PSHE and opportunities for PA sometimes suffering as a result. The difficulties of a crowded curriculum notwithstanding, schools are convenient sites at which to deliver information on making healthy choices [7], with their ability to reach almost the entire youth population. Recognising this, PA intervention programmes have often been delivered in school settings, supporting children in meeting the UK guidelines for at least 60 minutes’ participation in moderate-to-vigorous PA per day [8]. However, the aims of PA programmes often focus solely or predominantly on physical health, for instance the prevention of obesity [9].

The present study examined interventions in which PSHE and PA were delivered together in each of the weekly sessions, providing an interesting opportunity to explore two activities with potential wellbeing benefits from the perspectives of different stakeholders. That the interventions were delivered by external providers was another unusual feature worthy of investigation, and one which might address some of the issues associated with other interventions, such as limited sustainability when programmes are delivered by researchers; these interventions tend to take place over a period of time determined by the timescale and/or funding of the research project [10]. A common alternative is for researchers to train teachers in delivery, but in this case implementation compliance may suffer due to the imposition placed on teachers’ already limited planning time [11].

The purpose of this qualitative investigation was to explore the views of a range of stakeholders in relation to the interventions, and specifically: i) the role of the PA component; ii) children’s engagement in the sessions; and iii) intervention outcomes. Stakeholders were identified as children, parents, school staff and intervention staff, as key groups likely to hold an interest in the interventions. Through thematic analysis of the transcripts of interviews/focus groups with participants from these stakeholder groups, the aims of the investigation were met.

Reporting follows the consolidated criteria for reporting qualitative research (COREQ), a 32-item checklist for studies using interviews and focus groups [12].

## Method

### Interventions

The interventions were programmes in which PSHE information and PA opportunities were provided for primary school pupils for a six-week period. One PSHE topic (discrimination, drugs education, fitness and nutrition, self-esteem or racism) was communicated throughout the course, with each of the weekly sessions consisting of 30 to 60 minutes of classroom-based learning followed by a PA aspect of equal duration which took place either in the schoolyard or school hall depending upon the availability of facilities and weather conditions. Classroom messages were reinforced through the PA, for example by relating children’s successes to feelings of self-esteem or by allowing children to practise resisting peer pressure when making choices in the games, which were mainly team-based activities including variations on football, handball and tag-style games. Some schools opted for the PA aspect of the intervention sessions to count towards their weekly Physical Education (PE) provision, while others accommodated the intervention sessions alongside existing PE lessons.

The interventions were delivered by facilitators from football club foundations, organisations common to many professional football clubs in the UK as a means through which to provide PA- and education-related opportunities for their local communities.

### Participants

Invitations to participate in the study were extended to 13 schools in the North East of England whose Year 5 and Year 6 pupils (ages 9–11; final two years of primary school) were at that time taking part in an intervention. Schools were not incentivised to take part in the research. Five schools consented to participate, all with nearest residential postcodes in category 4 (Financially Stretched) or 5 (Urban Adversity) of the Acorn classification profile [13], indicating low socioeconomic status. The researcher (first author) visited each school to give a short verbal presentation to the classes about the study and to deliver information sheets and consent forms for pupils to take home to their parents; one of the schools unfortunately did not distribute these materials until it was too late for parents to respond and therefore no data were collected from this school. Across the remaining four schools, materials were distributed to a total of 148 pupils. Nine intervention staff and nine school staff were invited to participate in person or via email, four of the school staff being from an additional school (Acorn category 5) recruited specifically to increase the numbers in this participant group. Additional parents were not however sought because it became apparent from early interviews/focus groups that this group had limited knowledge of the interventions and it was considered unethical to pursue further recruitment.

The final sample consisted of all of the respondents with whom it was possible to make arrangements for data collection: 25 children (15 females), 5 parents (3 females), 6 members of school staff (all female) and all 9 members of intervention staff (1 female). The majority of the intervention staff were facilitators responsible for the delivery of the programmes, some having additional management responsibilities and one having a purely managerial role. School staff consisted of teachers, teaching assistants and one deputy head teacher. A total of 26 interview/focus group transcripts resulted from data collection.

### Instruments and procedures

Interviews and focus groups were conducted at the end of the interventions and followed semi-structured guides consisting of open questions pertaining to the pre-planned research questions. This gave participants freedom over their responses, with follow-up questions and prompts for clarification and elaboration employed where appropriate. To allow for wider discussion through comparison, questions were also asked about children’s non-intervention PSHE and PA experiences. A pilot study with parents, school staff and Year 5 children from a school not participating in the interventions was conducted in July 2016 to determine an appropriate duration for data collection and to assess the clarity of the questions on PA and PSHE and the level at which to pitch intervention-related questions. For example, in relation to engagement, children were asked: ‘What do you think about the programme?’ and in relation to outcomes, school staff were asked: ‘What do you feel the pupils have taken away from the programme?’

Data collection took place in quiet rooms either on school premises or at the workplaces of intervention staff, with interviews/focus groups ranging in length from 12 minutes and 23 seconds to 52 minutes and 20 seconds. Each one was audio-recorded and transcribed verbatim by the first author as soon as possible afterwards, with this author making reference to the notes they had taken immediately following data collection to provide a memory aid for the context of the discussions when transcribing. A member of school staff was allocated to sit in on three of the children’s focus groups and in the other four instances staff were in adjoining rooms and/or checked in throughout the session. Although the school setting might have affected participants’ behaviour (e.g. causing parents to respond in what they perceived to be an appropriate manner for parents of schoolchildren) [14], it was felt to be important for the researcher to meet the participants face-to-face at a familiar location to help establish rapport and allow them to feel comfortable sharing their experiences [15]. The researcher outlined her affiliation with the university but it is likely given the nature of the investigation that some of the participants perceived her to be associated with the intervention providers.

Parents, school staff and intervention staff were provided with participant information sheets and gave their written consent to participate in the study. In addition to their parents/guardians receiving information sheets, children were provided with age-appropriate information sheets. Children gave their written assent and their parents/guardians gave written consent for their participation. Approval to conduct the study was granted following review by the Faculty of Health and Life Sciences Research Ethics Committee at Northumbria University (submission ID: HLSNM030616a).

### Analysis

Thematic analysis was adopted due to its highly systematic, transparent approach to the identification of themes [16], allowing academic and non-academic audiences to understand this process in the interests of both research quality and clear communication to research users. The analysis progressed through six phases [17] and was conducted within a realist/essentialist paradigm, with themes being identified at a manifest level from the explicit reports of participants [18]. The research questions relating to engagement and outcomes were determined prior to data collection because findings in these areas were likely to lead to practical recommendations for future intervention provision; however, the inductive approach adopted for the analysis, coupled with the aim to provide a description of the entire data set, resulted in the evolution of an additional research question concerning the role of PA in the sessions, a topic of interest to the participants.

Transcripts were imported into QSR NVivo 10 (Copyright QSR International Pty Ltd., Melbourne, Australia) for coding and the identification of themes by the first author. A thorough, iterative process of refining the themes was then carried out by reviewing the data extracts assigned to each one, using the criteria of internal homogeneity and external heterogeneity for categories [19]. The validity of the themes was assessed by re-reading the transcripts to establish that the themes accurately represented the data set, and an in-depth account of each theme was written, including the main ideas it encompassed, comparisons and contrasts with other themes and how it provided insight into the research question(s). This was reviewed and agreed by the other authors. Direct quotes are provided in this report to illustrate participants’ experiences and substantiate the final themes [20].

Saturation of themes was approached using the Comparative Method for Themes Saturation (CoMeTS) [21]. The themes identified in each of the interviews/focus groups were compared four times: firstly in the order in which the interviews/focus groups were conducted, then in reverse order, and finally in two orders determined by random number generation. Themes shared with previous interviews/focus groups were noted, as were new themes. From the four comparisons, the greatest number of interviews/focus groups after which no new themes were found was four for the role of PA research question, eight for the engagement research question and five for the outcomes research question. Guest et al. [22] found that new themes emerged infrequently after 12 interviews but suggested this might differ for heterogeneous groups; however, in only one instance was a new theme identified in the final transcript for any of the research questions when broken down by stakeholder group, and this was for the parents, a group whose members knew little about the interventions and for which–likely as a result of this–there was great variety in the number of themes identified per transcript.

## Results

For clarity of reporting, the research questions are presented below as a series of headings beneath which their themes are discussed. The research questions are however best viewed in relation to one another, for instance with outcomes being reliant upon children’s engagement. A summary of the research questions and themes is provided in Table 1.

**Table 1:** Themes Identified for each of the research questions.

	Research Question		
	The Role of PA	Engagement	Outcomes
**Themes**	• Suitability for a range of children • Rest and reward • Reinforcement	• Enjoyment • Delivery by a non-teacher • Association with football club	• Psychosocial wellbeing • Physical wellbeing

Quotes are followed by a participant’s stakeholder group (C: child, IS: intervention staff, P: parent, SS: school staff), transcript number and the PSHE topic(s) covered in the programme(s) upon which they were commenting (DI: discrimination, DR: drugs education, FN: fitness and nutrition, SE: self-esteem, RA: racism).

### The role of PA

This research question emerged from participants’ discussion of the two components of the intervention sessions. PSHE delivery through classroom-based learning and PA was unfamiliar but valued: ‘It’s a really good balance’ (P3,SE). Three themes were identified to explain the positive perception of PA in the sessions.

#### Suitability for a range of children

Firstly, it was felt that the inclusion of PA alongside classroom work made the sessions appealing to a range of children: ‘some people… sometimes enjoy, like, inside the classroom with [facilitator] as he makes it fun… And then quite a lot of the people, like, aim to be good at sport and they really enjoy the little games’ (C6,SE). The two components also provided different modes of learning: ‘some pupils get benefit from the course within the classroom. Some benefit more within… the PE side. And some for both’ (IS1,DR/SE/RA).

#### Rest and reward

Secondly, and supporting children’s engagement in the sessions, PA was perceived as a break from and/or positive reinforcement for participation in the classroom activities: ‘you felt like you’ve done your work and, like, now’s the time to just have some fun outside. And it felt like a reward for being in the classroom’ (C1,DR). At the same time, one of the school staff indicated that the PA might act as *negative* reinforcement by helping to relieve some of the ‘tension’ resulting from the issues covered on the racism programme (SS1,RA).

#### Reinforcement

Finally, as intended, PA was reported to be a useful conduit via which facilitators could reinforce intervention messages:

for classes which are quite well-behaved and not a lot is said within the classroom, when it comes to the PE side they tend to open up more. And then you may get one or two things which are said… generally in the heat of the moment, which are a little bit unkind… but… you relate it back to what was spoken about in the classroom. You know, “Do you know by saying that… you could be affecting their self-esteem?” (IS1,SE)

Children and school staff generally understood the link between the PA and classroom components of the sessions: ‘it was the sexism [classroom session] yesterday. But then [in the PA] they were saying… sometimes you’ll have, like, an all-girls team and an all-boys team, and we were mixing them yesterday’ (SS5,DI). However, in some instances clarification appeared to be required: ‘some weeks it’s linked and then others it’s, like, not really got anything to do with it’ (C5,SE).

### Engagement

Three themes pertaining to children’s engagement in the sessions were identified and are presented below from most to least prevalent. It is however also of note that while the role of home support for healthy behaviours including PA was well recognised, interestingly, none of the parents reported knowing very much about the interventions. They had therefore been unable to support their children’s engagement and reinforce the messages being taught. Suggestions were made for the inclusion of ‘anything within the… programme where you could, say, do some activities at home with… your parents so we… understand a little bit more’ (P1,DR).

#### Enjoyment

Children very much enjoyed the interventions: ‘overall it was just really fun. We did loads of fun things in the classroom and out there, as well’ (C4,DR); ‘She loves it… She comes in and she’s buzzing when she’s had it’ (P3,SE). Classroom tasks were designed to actively involve the children in order to make them enjoyable and engaging: ‘lots of activity, lots of involvement of the children, like letting them almost lead it into, kind of, getting them to be engaged’ (SS4,FN/DI). Active involvement extended to promoting one another’s engagement; comments regarding non-intervention PA indicated that children enjoyed supporting others–‘Now he’s in Year 6, he’s encouraging all the Year 1s and the Year 2s’ (P1,DR)–and facilitators used this inclination within the interventions: ‘“your mate over there… There’s something up, will you just have a chat with him?’’’ (IS1,DR/SE/RA).

The variety of games also contributed to enjoyment: ‘it’s always really fun ‘cause you’re never doing the same thing in two weeks’ (C6,SE). However, mention of variety was more common in relation to non-intervention PA, in which participants reported that children enjoyed taking on new challenges and developing competencies through ongoing learning, with different children having different ‘hidden talents’ (C1,DR) that they may find through variety in PA participation.

On a practical level, enjoyment was supported by being outside ‘because it’s a bigger space than the hall. The hall’s quite a tight squeeze for, like, all the class’ (C5,SE). However, it was not always possible for PA to take place outside due to weather conditions and rotas for school facilities.

#### Delivery by a non-teacher

The status of intervention staff as visitors to the school was another factor contributing to children’s engagement, with many participants noting that ‘if there’s other people coming in, the children see it as a treat… and something special’ (SS1,RA). The informal mode of delivery and the characteristics of the facilitators were felt to help: ‘he’s really, like, funny and he’s fun’ (C7,RA).

Intervention staff identified that in contrast to many primary school teachers, ‘a lot of our delivery staff are men’ (IS7,FN/DI), and that this could promote or discourage engagement for different individuals. They also described that working with school staff was important for engagement: ‘When you get a teacher who joins in, it’s like a positive role model for the kids and they think, “Well… if miss is doing it, and she’s listening… then I’ll do it’’’ (IS6,FN/DI).

#### Association with football club

Intervention staff perceived their associations with their football clubs to have a positive influence on engagement, for instance by providing a ‘hook’ for the children (IS4,DR/SE/RA) and by helping the facilitators to build rapport: ‘It’s just a… good talking point to start with… It’s something as simple as… “Who’s seen the score?”… whereas if you weren’t from [football club] you’d have to find some common ground’ (IS5,FN/DI). Furthermore, facilitators were seen to be role models: ‘If they come in here… wearing a [football club] jacket and top, especially the more difficult boys look up to them and will listen’ (SS6,FN/DI); children noted that if they were to design their own healthy lifestyle programme, ‘I would have–‘cause… this’ll make the boys happy–some football players’ (C7,RA), and that when they received PA sessions from external providers it ‘feels like they’re role models to you’ (C1,DR).

On the other hand, girls in particular were sometimes deterred by an initial perception that the interventions would be football-orientated. Intervention staff were very aware of this potential drawback to the football club association:

the first lesson it’s something I would always say… it’s not going to be all football. It’s just going to be fun games. …there’ll be someone rubbing their hands, going, “I can’t wait for this,” ‘cause they love football. But there’ll be one or two sitting there thinking, “Ooh, I’m not sure about this.” They don’t know what to expect… Are they going to have to score goals? …But we just, sort of, relax them and say, “Look, they’re… just fun. Don’t worry about it.” Everyone buys into it. (IS4,DR/SE/RA)

### Outcomes

A number of benefits to children’s participation in the interventions were identified, all of which broadly pertained to two themes: psychosocial wellbeing and physical wellbeing. The chance to observe facilitators’ PA delivery was additionally recognised by teachers as an opportunity ‘for my own CPD [continuing professional development], as well… if you’re going to be taught by the best for football, then get them in, I think, so… we can all learn together’ (SS3,DI/FN).

#### Psychosocial wellbeing

Overall, participants were very positive about the interventions and felt that they improved children’s knowledge of PSHE topics. For example, children who had taken part in the drugs programme listed many facts about the types and effects of drugs and demonstrated an understanding of how drug use can begin:

it said that there was people that were curious or they were just bored and they’d tried alcohol… or they tried drugs. If you try them… your personality could change… and then you could get addicted to it and it could just change, like, your lifestyle. Your career. (C3,DR)

It was envisaged in the interventions’ aims that PSHE knowledge would help children in practical ways, such as by assisting them in making healthy food and activity choices and by giving them the skills to raise their own/others’ self-esteem. There were some examples of this having taken place over the duration of the programmes, for instance in relation to the self-esteem course one parent reported: ‘[her brother]’ll do something, and she’s like, “You’re doing really good, there!’’’ (P3,SE). Participants felt that such knowledge and skills would be beneficial in the future, and especially in the transition to secondary school, but it was not possible for them to comment on long-term outcomes.

From the accounts of three of the intervention staff, five of the school staff and one of the parents, the interventions–and importantly not just the self-esteem programme–were seen to be beneficial for children’s confidence/self-esteem; self-esteem was in all but one instance mentioned in the context of confidence so in order to reflect the data no academic distinction is drawn between the two. Although none of the children reported feeling more confident themselves, the adult participants had noted changes in pupils’ behaviour: ‘over the weeks they’ve relaxed… even the quiet ones’ll put their hand up and have a go. And they’re not afraid that they get something wrong. … and… they’re putting their effort in outside [in the PA]’ (SS5,DI).

Finally, the interventions–and particularly the PA component of the sessions–promoted teamwork: ‘[the facilitator says it’s] just about having fun and working together as a team’ (C7,RA). Similar to the application of PSHE knowledge, while children were felt to be learning about teamwork, there were few examples of observed improvements; however, one of the intervention staff reported that ‘by the end of the… course, everyone’s, sort of, in harmony, and they’re all supporting each other… you can see it developing as the weeks go by’ (IS4,DR/SE/RA).

#### Physical wellbeing

Intervention staff for the fitness and nutrition programme reported that ‘from results that we do carry out in terms of the activity, [the children] show improvements that they’ve increased their fitness levels and activity levels, so it’s doing its job’ (IS9,FN). School staff whose pupils had participated in this programme–and were now taking part in the discrimination programme–had noticed effects for PA participation outside of the programme sessions: ‘the girls especially… they’ve started doing different things, and they’re joining in more on a playtime, as well. Where they would tend to be in their little huddles, they’re… picking up a skipping rope, or… the basketballs’ (SS5,FN/DI). There had been notable health benefits for one pupil in particular:

she struggled with her confidence and she was also struggling with her weight? … And we talked to her about the things that, like, the [facilitators] had said are good to eat. … And we’ve spoken about the exercise things. And she actually came in the other day and we said, “You need a new jumper ‘cause that one’s too big.” So, you can actually see that she *has* taken it in, but her confidence has soared, as well. (SS5,FN)

Adult participants less frequently noted the physical health benefits of participation in other programmes, but children recognised that ‘We got exercise!’ (C1,DR) and that ‘you get to keep fit and healthy when you do the physical activity’ (C5,SE). One of the parents did however identify increased PA as an outcome from the self-esteem programme:

I know it’s encouraging [my daughter] more, in that she wanted to go out and do more. Like, more sporty stuff. ‘Cause she’s not normally sporty. She hates it! I mean, she would hate walking; where now, she wants to come out with the dog. (P3,SE)

In a similar pattern, it was only intervention and school staff with experience of the fitness and nutrition and discrimination programmes that mentioned the potential for the PA aspect of the interventions to motivate or tire children for any learning activities that followed, although a handful of participants referred to these effects following other forms of PA at school. One member of school staff felt particularly strongly that, ‘Once they’ve got rid of that energy, they can focus’ (SS6,DI/FN), but at the same time intervention staff reported a preference across schools for the interventions to be delivered in the afternoon.

## Discussion

Overall, the interventions were viewed very favourably across all of the stakeholder groups. The classroom and PA components of the sessions were felt to work well together, the children were reported to be highly engaged and there were perceived benefits of participation for pupils’ psychosocial and physical wellbeing.

### The role of PA

The role of PA in the interventions was explored in response to participants’ interest in the unusual format of the sessions: classroom learning followed by physically active games. Participants from all groups identified that PA played a role in children’s engagement by appealing to those who may not have invested in a purely classroom-based programme but were enthused by the games, or vice-versa. Engagement was thought to be further promoted via children’s perception of the PA as a reward for participating in the classroom activities, and one member of school staff suggested that PA participation possibly provided relief following classroom study of demanding PSHE topics. PA might therefore stimulate engagement through both positive and negative reinforcement, providing a reward for children’s work and an escape from any discomfort to encourage their continued participation [23].

In addition, PA was thought to support intervention outcomes, with intervention staff recognising that the different components of the sessions might meet different children’s preferred styles of learning [24], although experimental evidence for an interaction between learning styles and instructional methods for educational outcomes is limited [25]. However, PA was not used simply to reiterate messages; taking part in the games encouraged children to ‘open up more’ (IS1), allowing them to experience behaviours they had discussed in the classroom [26] and put their learning into practice. In a small number of cases the children had unfortunately failed to appreciate the link between the classroom and PA components and it may be that some PSHE topics or programme messages more readily lend themselves to expression through PA. For example, for the heart rate topic children were able to measure their heart rates before and after exercise, meaning ‘sometimes… it’s easy… But some of them are… obviously, with regards to food labelling… I think it would be nigh on impossible’ (IS5,FN).

### Engagement

Consistent with the above discussion, the leading factor behind children’s engagement in the interventions appeared to be their enjoyment, and in particular their enjoyment of the PA component. The reasons for children’s enjoyment seemed to broadly map onto self-determination theory [27], which proposes that intrinsic motivation (taking part in an activity because it is inherently enjoyable or interesting) is supported by environments stimulating feelings of autonomy, relatedness and competence [28]. Facilitators made efforts to promote autonomy and relatedness by fostering a sense of ownership over the classroom activities and games and by encouraging peer support throughout the programmes, while children further touched upon relatedness in noting that they liked the facilitators. Children and intervention staff also referred to the variety of games contributing to the appeal of the interventions to a range of children, but variety was more frequently cited in terms of non-intervention PA, where it was linked with taking on new challenges and different children excelling in different activities. It is possible that including more PA-based challenges and opportunities for skill development would better address the ‘competence’ aspect of self-determination theory; however, there were few concerns over children’s engagement levels and negotiating access to more spacious indoor facilities in the event of poor weather would be likely to have a greater impact on enjoyment.

As intended, the association of the interventions with football clubs appeared to contribute to children’s engagement. The facilitators’ status as role models through children’s familiarity with the football clubs also made them potentially more persuasive at changing attitudes and behaviour [29], strengthening intervention outcomes. A possible barrier to initial engagement was recognised for children who did not enjoy football, in that they might anticipate the PA component being football-centric, but this misperception was addressed early in the programmes and was not reported to cause any further difficulties for participation.

A key modification to the interventions advocated by participants, and one which would likely support children’s engagement *and* intervention outcomes, was the addition of parental involvement. Parents knew little of the interventions and could not therefore express an interest in this aspect of their children’s education, such interest having been related to the value placed on learning by a secondary school sample [30]. Neither could parents reinforce the programme messages, despite participants recognising the role of the home environment in promoting healthy behaviours and evidence existing for parents’ influence on, for example, children’s self-esteem [31] and intention to smoke/initiation of smoking in adolescence [32–33]. Suggestions were made by participants for the introduction of homework activities for children to complete with their parents as a method of communicating programme content.

### Outcomes

All of the participant groups suggested that children’s participation in the interventions was beneficial for their psychosocial wellbeing, specifically in terms of their knowledge of PSHE topics, their confidence/self-esteem (the only subtheme not to be noted by all groups as it was missing from the children’s accounts) and their appreciation of teamwork. Increased confidence was felt to be underpinned by a number of factors including an improved understanding of how to cope with everyday issues, being entrusted with responsibilities to help others in the intervention sessions and experiencing increased peer support; factors which have been found to promote self-esteem and a sense of being needed in youth/young adult populations [34–36]. Due to the concrete nature of their content, increased knowledge may be more apparent for some programmes (e.g. drugs education) than others (e.g. self-esteem), making their learning easier for children to express. The main difficulty, however, lies in assessing whether an increase in knowledge translates to children making positive choices later in life; knowledge has been associated with positive choices regarding food for this age group [37], and the interventions do appear to provide learners with “the knowledge and skills necessary to make safe and informed decisions” [2], but the accounts in this study were speculative about long-term effects because follow-ups have yet to be conducted. Similarly, children were thought to have acquired an understanding of teamwork but there were few tangible examples of this having been demonstrated in the limited duration between intervention participation and data collection.

Important as they are, the above gains might for parents and school staff have overshadowed the physical wellbeing outcomes of participation in programmes concerning topics other than fitness and nutrition. Although the PA component reinforced the classroom content, supported children in reaching the UK guideline of at least 60 minutes of moderate-to-vigorous PA per day [8] and may have also independently enhanced characteristics and skills such as self-esteem, self-discipline and teamwork [5], opportunities to highlight PA-specific benefits may be being missed in non-fitness and nutrition programmes. Furthermore, investigations of the effects of the interventions on schoolwork would be valuable to help school staff decide when to accommodate them in their timetables for the greatest (or least detrimental) impact of post-session energy levels in the immediate term and to assess whether there are improvements in academic achievement over the longer term, as would be anticipated from previous social and emotional learning programmes [4].

### Limitations

One of the strengths of the study was its inclusion of a range of stakeholders, helping to construct a picture of the interventions from the viewpoints of a number of groups with an interest in them. However, only a limited number of schools invited to participate in the study accepted and from those schools only a limited number of individuals invited to participate responded. It may be that these particular schools/participants were all keen to provide feedback due to their apparently positive experiences of the interventions. Future research may benefit from purposefully seeking contrary views for a more complete picture; for example, participants identified that some children were concerned at the beginning of interventions that the programmes would be highly football-orientated, and children with these concerns could be asked for their input to explore this issue. Furthermore, the sample of school staff was all female and insights from male members of staff may have complemented the data collected, although the participants reported that the majority of primary school employees are female and the sample is reflective of this. Similarly, one of the members of school staff was a deputy head teacher but the inclusion of additional participants at the school leadership level might allow for greater depth of analysis regarding initial decisions to take up interventions. Wider impacts of schools’ involvement in interventions might also be a point of discussion with school leaders, for instance whether there are likely to be/have been changes made to teaching practice based on observation of intervention delivery and pupils’ responses to intervention content, staff, etc.

In addition to the above limitations concerning the sample, it is recognised that a level of socially desirable reporting is likely given the health-related topic matter and the potential for school and intervention staff to feel that their responses would reflect upon their school/organisation. Many of the participants also speculated positively on the long-term outcomes of intervention participation but it must be reiterated that long-term follow-ups have not yet been conducted and the data collected simply represent stakeholder expectations. Future research may wish to explore children’s long-term outcomes from intervention participation.

### Recommendations for intervention development

An important aspect of any intervention research is that recommendations are made for future development of the programmes in order that research users such as schools, pupils and intervention staff can benefit from the findings. Recommendations must also be feasible, for example taking into account cost and the practicalities of delivery in a school environment.

Unfortunately, stakeholders’ leading proposal for intervention development would incur additional costs, with participants suggesting an increase in duration from the current six-week courses to better support pupil outcomes. A more economical approach may be for intervention or school staff–who commented on the CPD benefits of observing facilitators for their own future PA delivery–to run follow-up PA-only sessions to remind pupils of programme messages; an approach recommended to help UK youths maintain healthy activities following an obesity programme [38].

At present, UK primary schools are able to pay for interventions of this type using the PE and Sport Premium provided by the government [39]. Aware that one of the requirements of the premium is for schools to ensure that improvements made to their PA provision are sustainable, some of the intervention staff noted that they were considering adding a more formal CPD component to the interventions, allowing staff from across a school to learn how to enhance their current and future PE lessons. It must however be noted that one of the themes identified for children’s engagement in the intervention sessions was that they were delivered by a non-teacher, with children responding favourably to visitors to the school, and therefore if school staff were to take on intervention delivery themselves their pupils may not engage to the same degree. It is recommended that while teachers receive training from the football club foundations to enhance their delivery of PE lessons and PA-only intervention follow-up sessions, the interventions themselves continue to be delivered by foundation staff. In the case of the PE and Sport Premium being discontinued and cost becoming prohibitive for schools, intervention staff may consider seeking funding from organisations such as charities, as they reported they have done in the past, to support them in delivering healthy lifestyle information to schoolchildren.

The major barrier to school uptake of the interventions was the difficulty of accommodating the sessions in a busy school timetable. While the interventions help schools to meet the national curriculum requirement to provide PSHE opportunities for their pupils [1], and while some schools count the PA aspect of the intervention sessions towards their weekly PE provision, there is still seemingly a the prioritisation of core curriculum subjects [6], particularly in the morning. Academic achievement was referenced in relation to the interventions in very few of the transcripts, possibly indicating a lack of awareness of the positive relationships between wellbeing and academic achievement [4]. As school staff are more likely to welcome changes which address learning and teaching problems they are experiencing [40], such as meeting attainment levels in core subjects, research into the academic outcomes of intervention participation (including any impact of post-session energy/motivation levels on learning) is warranted to support school uptake. School staff also referred to the regular weekly scheduling of the sessions as potentially problematic for intervention uptake as it was possible for one or more of the six sessions to conflict with events such as school trips. Greater flexibility was therefore requested, but this is unlikely to be feasible due to the commitments of football club foundation staff to intervention delivery in other schools and the difficulties of securing flexible access to appropriate school facilities for the PA session around the needs of other classes. Session content is however flexible enough to rearrange activities in order to minimise loss of learning if classes are unable to participate in all of the sessions, and requests from school staff can also be accommodated if they feel their pupils would benefit from input on a particular PSHE topic.

Finally, parents requested that they were informed of intervention content in order that they could reinforce messages and support long-term outcomes for their children outside of the school setting. As described earlier in the discussion, children’s engagement in the intervention sessions might also be enhanced were their parents informed enough to be able to express an interest in the programmes. Although cost was a factor for some of the suggestions offered to address this issue, for instance in sending information sheets about the interventions to parents, the addition of homework activities for children to complete with their parents should incur no additional costs and the completed work would serve as evidence that parents had been successfully informed. It is recommended that homework activities are added to the programmes which communicate not only PSHE content but how this is being promoted through PA participation during the intervention sessions and how parents can reinforce messages consistent with those of the interventions over the long term. This would be anticipated to increase the impact of the interventions–and to raise awareness of the PA component and the potential benefits of the interventions for physical wellbeing–although again further investigation is required to explore long-term outcomes and whether parental involvement leads to any greater effects than intervention delivery as it currently stands.

## Conclusion

This study provided insights from the perspectives of children, parents, school staff and intervention staff into interventions delivering PSHE topics through a combination of classroom learning and physical activity.

One of the main findings was that children engage in the interventions due to their enjoyment, particularly of the PA component of the sessions. Although the PA may be perceived as a reward for engagement in classroom learning, overall it appears that intervention participation has some degree of internal motivation [27], and it is reasonable to conclude that children might as a result be more receptive to programme messages than if they lacked personal control over their participation. The main benefits of the interventions were perceived to be improvements in knowledge, confidence/self-esteem, teamwork and–for the fitness and nutrition programme in particular–physical wellbeing, but no information currently exists for long-term outcomes and further investigation is recommended.

On the whole, the current data indicate that the interventions were valued by stakeholders. Some suggestions were however offered for development of the programmes, including the addition of follow-up sessions and activities to be completed with parents, helping to support children’s outcomes through reinforcement of intervention messages over longer periods and in the home environment.
